# Microcephaly Gene *Mcph1* Deficiency Induces p19ARF-Dependent Cell Cycle Arrest and Senescence

**DOI:** 10.3390/ijms25094597

**Published:** 2024-04-23

**Authors:** Yi-Nan Jiang, Yizhen Gao, Xianxin Lai, Xinjie Li, Gen Liu, Mingmei Ding, Zhiyi Wang, Zixiang Guo, Yinying Qin, Xin Li, Litao Sun, Zhao-Qi Wang, Zhong-Wei Zhou

**Affiliations:** 1 Shenzhen Key Laboratory for Systems Medicine in Inflammatory Diseases, School of Medicine, Shenzhen Campus of Sun Yat-sen University, Sun Yat-sen University, Shenzhen 518107, China; jiangyn23@mail2.sysu.edu.cn (Y.-N.J.); lixin253@mail.sysu.edu.cn (X.L.); 2 Shenzhen Key Laboratory of Pathogenic Microbes and Biosafety, School of Public Health (Shenzhen), Shenzhen Campus of Sun Yat-sen University, Sun Yat-sen University, Shenzhen 518107, China; gaoyzh8@mail2.sysu.edu.cn (Y.G.); sunlt@mail.sysu.edu.cn (L.S.); 3 State Key Laboratory of Microbial Technology, Shandong University, Qingdao 266237, China; zhao-qi.wang@sdu.edu.cn

**Keywords:** microcephaly, MCPH1, cell cycle, senescence, p19ARF, e2f1

## Abstract

*MCPH1* has been identified as the causal gene for primary microcephaly type 1, a neurodevelopmental disorder characterized by reduced brain size and delayed growth. As a multifunction protein, MCPH1 has been reported to repress the expression of TERT and interact with transcriptional regulator E2F1. However, it remains unclear whether MCPH1 regulates brain development through its transcriptional regulation function. This study showed that the knockout of *Mcph1* in mice leads to delayed growth as early as the embryo stage E11.5. Transcriptome analysis (RNA-seq) revealed that the deletion of *Mcph1* resulted in changes in the expression levels of a limited number of genes. Although the expression of some of E2F1 targets, such as *Satb2* and *Cdkn1c*, was affected, the differentially expressed genes (DEGs) were not significantly enriched as E2F1 target genes. Further investigations showed that primary and immortalized *Mcph1* knockout mouse embryonic fibroblasts (MEFs) exhibited cell cycle arrest and cellular senescence phenotype. Interestingly, the upregulation of p19ARF was detected in *Mcph1* knockout MEFs, and silencing *p19Arf* restored the cell cycle and growth arrest to wild-type levels. Our findings suggested it is unlikely that MCPH1 regulates neurodevelopment through E2F1-mediated transcriptional regulation, and p19ARF-dependent cell cycle arrest and cellular senescence may contribute to the developmental abnormalities observed in primary microcephaly.

## 1. Introduction

Microcephaly Primary Hereditary (MCPH) is a rare autosomal recessive disorder characterized by a significant reduction in the frontal head circumference and mild to moderate intellectual disability. The prevalence of all forms of microcephaly that exist from birth has varied from 1 in 30,000 to 1 in 250,000 newborns worldwide [1]. The patients exhibited thinning of the cerebral cortex, often accompanied by delayed growth, epilepsy, and other symptoms [2]. Microcephaly is present at 32 weeks of gestation and can be observed at birth, with no significant improvement after birth [2]. Therefore, studying MCPH can reveal the pathological mechanisms leading to this condition and the normal developmental processes.

The *MCPH1* gene encoding Microcephalin, also known as *BRIT1* (BRCT-repeat inhibitor of TERT expression 1), was the first reported to cause MCPH type 1 (MCPH1, OMIM251200). To date, 12 different bi-allelic pathogenic variants in the *MCPH1* gene have been identified as the cause of MCPH [3]. To explore the causal role of *MCPH1* in microcephaly, several *Mcph1* knockout mouse models have been generated [4]. These mutants successfully reproduced the characteristics of primary microcephaly caused by the human *MCPH1* mutation. Furthermore, we have previously generated the *Mcph1* null mutant mouse model by targeted deletion of *Mcph1* exon 4–5 (Mcph1^tm1.1Zqw^) [5]. Gruber et al. found that disruption of *Mcph1* can cause microcephaly in the mouse model due to a premature transition of neural progenitor cells from symmetrical to asymmetrical division [5].

The most current research has focused on the mechanism of *MCPH1* causing microcephaly while ignoring another phenotype in MCPH1 patients. People with the *MCPH1* mutation are characterized by significantly reduced head circumference and shorter height [6,7,8]. In some patients, short stature is a variable feature of human MCPH1 [9]. The same phenotype is found in monkeys, and female *MCPH1^mt/mt^* monkeys have been shown to exhibit a slightly shorter stature than wild-type monkeys. The weight of the *MCPH1^mt/mt^* monkey was only ~65% compared with wild-type animals before six months of age and below average (−2 to −8 SD) during the first year of life [10]. The first *Mcph1^−/−^* mouse model was established by deleting exon 2 of mouse *Mcph1* using the Cre/loxP system. These mice showed no significant microcephaly but did show delayed growth and weighed only 80% of wild-type litter pups [11]. Zhou et al. found that *Mcph1* mutant mice had an approximate 20% reduction in body weight (at P0, P21, and P180) [12]. However, the reason for delayed growth caused by *MCPH1* dysfunction is unclear.

Microcephalin, identified initially as a transcription suppressor of h*TERT*, can directly bind to the proximal promoter of the h*TERT* gene to inhibit h*TERT* promoter activity, resulting in the reduction in h*TERT* expression and telomerase activity [13], indicating MCPH1 itself plays a role in regulating transcription processes. Moreover, MCPH1 can interact with the chromosomal remodeling SWI/SNF complex to relax chromosome structure. Chromosome remodeling is closely related to transcription, suggesting that MCPH1 may play a role in transcriptional regulation [14]. Furthermore, human as well as rhesus macaque MCPH1 directly binds to E2F1 to regulate the transcriptional activity of E2F1 on the promoters of its target genes, such as *Chk1*, *Brca1*, *CyclinE1*, and *Cdkn2a* [15,16]. All these suggest that MCPH1 plays a role in transcription regulation. However, whether MCPH1 regulates the fate of neural stem cells and cerebral cortex development through, especially, E2F1-mediated transcriptional activity is entirely unknown. 

In this study, we found that delayed growth was observed after the deletion of *Mcph1* during the early embryonic development stage (E11.5). RNA-seq analysis of brain samples showed that *Mcph1* was unlikely to affect neurodevelopment through E2F1-related transcription factor activity but may affect neurodevelopment through cell cycle inhibitors such as *Cdkn1c*. Then, we found that *Mcph1-KO* primary MEFs exhibited slow cell proliferation. Further investigation found that *Mcph1-KO* induced cell cycle arrest at the G1 phase, leading to premature cell senescence and upregulating p19ARF (encoded by *Cdkn2a*) levels. Interestingly, the *Mcph1* knockout immortalized MEFs lacking *p19Arf* exhibited near-normal cell cycle and growth compared to the controls. These results suggested that deficiency of MCPH1 may indirectly cause neurodevelopmental abnormalities and delayed growth through the cell cycle rather than transcriptional regulation function.

## 2. Results

### 2.1. Mcph1 Knockout Results in Delayed Growth Early in the Embryonic Stage

**Figure 1:**
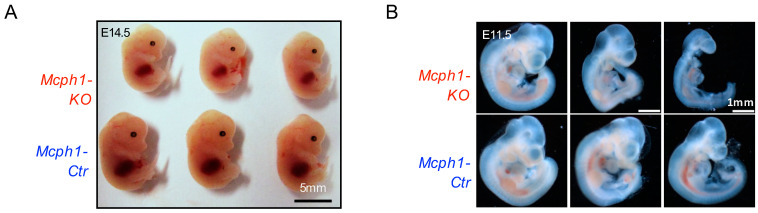
*Mcph1-KO* embryos exhibit delayed growth during an early stage. (**A**) Images of the whole body of control and *Mcph1-KO* mice at embryonic period 14.5 (E14.5). Scale bar, 5 mm. *Mcph1-Ctr* is the control group, and *Mcph1-KO* is the *Mcph1* knockout group. (**B**) Images of the whole body of control and *Mcph1-KO* mice at embryonic period 11.5 (E11.5). Scale bar, 1 mm.

Zhou et al. previously reported that *Mcph1* knockout mice were smaller than their littermates at the postnatal day 1 (P1) [12]. *Mcph1-KO* mice lose weight after birth, but size before birth was not known. We found that *Mcph1-KO* mice were smaller at E14.5 (embryonic day 14.5) compared to littermates (Figure 1A). Then, we found that the same phenotype was already present at E11.5 (Figure 1B). These findings demonstrated that *Mcph1* knockout results in delayed growth and brain development disorders that are already present early in the embryonic stage. 

### 2.2. Mcph1 Deletion Affected Metabolism and Development Processes

**Figure 2:**
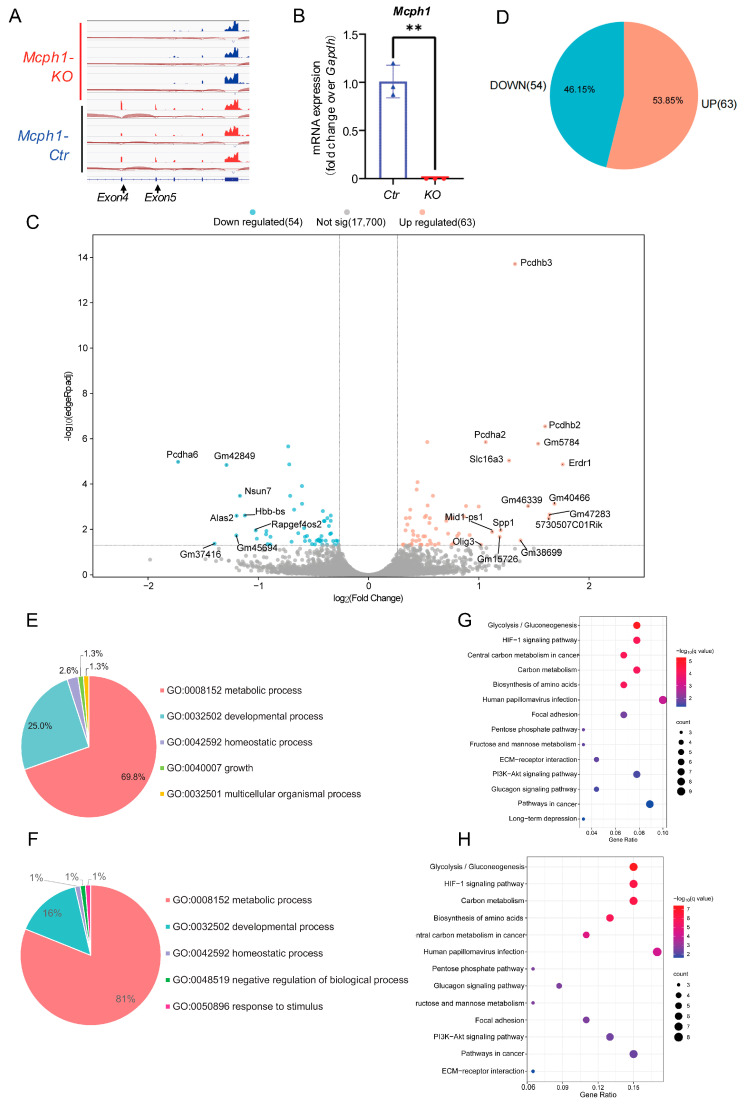
The metabolism and development processes are mainly affected by *Mcph1* disruption. (**A**) Gene mapping analysis of mice in the control group and *Mcph1-KO* group. The arrow refers to the location of exons 4 and 5. (**B**) The content of *Mcph1* in *Mcph1-Ctr* and *Mcph1-KO* mice was detected by RT-qPCR. The horizontal coordinate represented the group, the vertical coordinate represented the relative expression of *Mcph1* mRNA, and *Gapdh* was used as a reference. **, *p* < 0.01. (**C**) The volcano plot of the *Mcph1-KO* group against the control group. DEGs with a fold change greater than 1.2 and adjusted *p* value of less than 0.05. Red represents upregulated DEGs, and blue represents downregulated DEGs. Gray indicates genes that are not statistically significant. (**D**) The number and proportion of genes in DEGs. Red shows the proportion of upregulated DEGs, and blue shows downregulated DEGs. (**E**) Classification of biological processes of all DEGs. The metabolic process accounted for 69.8%, and the developmental process accounted for 25.0%. (**F**) Classification of biological processes of upregulated DEGs. The metabolic process accounted for 81%, and the developmental process accounted for 16%. (**G**) Enriched KEGG pathways of all DEGs. KEGG analysis shows the enriched items in all DEGs. (**H**) Enriched KEGG pathways of upregulated DEGs. KEGG analysis shows the enriched items in the upregulated DEGs.

To further investigate the role of *Mcph1* in brain development, we isolated the brains of E14.5 embryos and performed RNA sequencing (RNA-seq). *Mcph1-KO* mice produce the termination code in advance by knocking out exons 4 and 5 of *Mcph1*, rendering the MCPH1 protein unable to express [5,12]. Gene mapping analysis of the sequencing results showed that the expression peaks of exons 4 and 5 were detected in the control group but not in the *Mcph1-KO* group (Figure 2A). RT-qPCR verified a decrease in *Mcph1* expression in the cerebral cortex of the *Mcph1-KO* mice (Figure 2B). 

We used DESEQ2 analysis to calculate differential expression levels between the *Mcph1-Ctr* and *Mcph1-KO* groups and visualized the results using volcano maps (Figure 2C). There were 117 significantly differentially expressed genes (DEGs) (*p* adj < 0.05), with 63 upregulated and 54 downregulated (Figure 2D). The significant biological process (BP) terms generated by Gene Ontology (GO) enrichment of all the DEGs mainly include metabolic process, developmental process, homeostatic process, growth, and so on (Figure 2E). The metabolic process, developmental process, and homeostatic process of the BP were obtained from the upregulated DEGs (Figure 2F), and no BP items were obtained from the downregulated DEGs (*p* adj > 0.05). Furthermore, according to the KEGG pathway enrichment analysis, all the DEGs and upregulated DEGs were significantly enriched in more than 10 signaling pathways (*p* < 0.05), mainly involving metabolism-related pathways such as glycolysis/gluconeogenesis, HIF-1 signaling pathway, central carbon metabolism in cancer, carbon metabolism, and biosynthesis of amino acids (Figure 2G,H). All these indicated that the depletion of *Mcph1* affects mainly metabolism and development processes.

### 2.3. The DEGs Caused by Mcph1 Depletion Are Not Enriched for Transcription Factor E2F1

**Figure 3:**
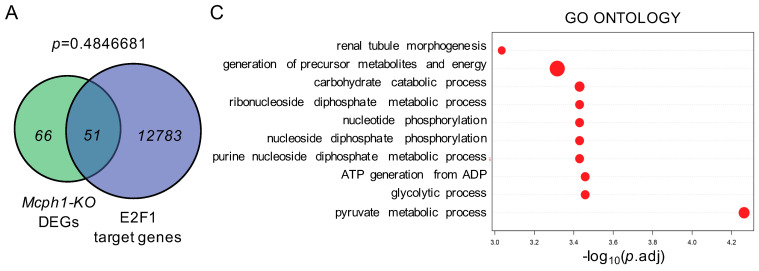

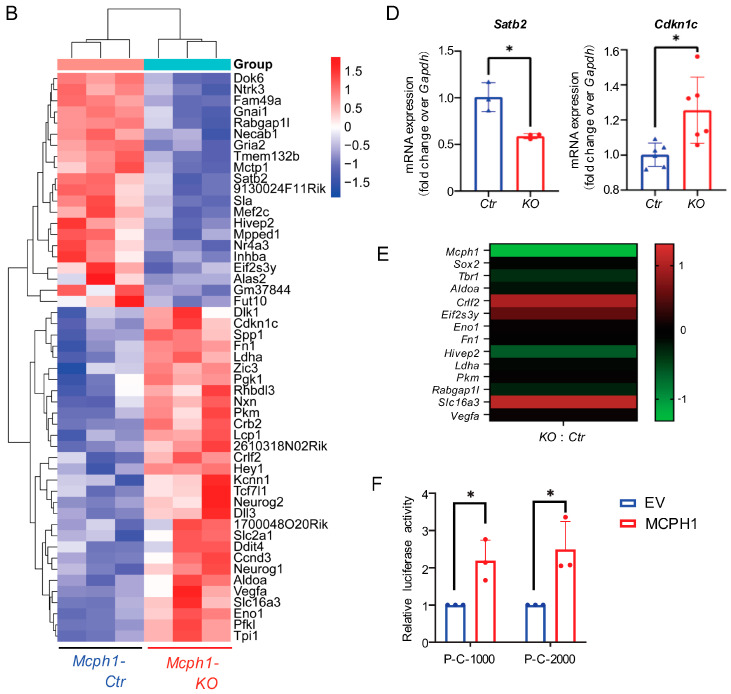
Analysis of the intersection of *Mcph1-KO* DEGs and E2F1 target genes. (**A**) The Venn diagram represents the intersecting genes between E2F1 target genes and *Mcph1-KO* DEGs. There were 12,834 E2F1 target genes, 117 *Mcph1-KO* DEGs, and 51 overlapping genes. *p* > 0.05, no statistical significance. Statistical analysis was performed using Fisher’s exact test. (**B**) Correlated intersecting genes heat map. The abundance of 51 genes shared by all *Mcph1-KO* DEGs and E2F1 target genes was shown. (**C**) Biological processes associated with intersecting genes. GO analysis was performed on intersecting genes, and the top 10 biological processes are shown on the left. (**D**) Expression verification of intersecting genes related to neurodevelopment, such as *Satb2* (encoded for special AT-rich sequence-binding protein 2) and *Cdkn1c* (encoded for p57KIP2). The expression of intersecting genes in control and *Mcph1-KO* groups was detected by RT-qPCR. *, *p* < 0.05. (**E**) Heat map of RT-qPCR test results for neurodevelopment-associated intersecting genes. (**F**) The effect of MCPH1 overexpression on *Cdkn1c* promoter activity was detected by double luciferase assay. *, *p* < 0.05. P-C-1000 and P-C-2000 were different lengths of *Cdkn1c* promoter regions.

MCPH1 can bind to the SWI/SNF complex [14]. MCPH1 can interact with the core proteins of this complex, including BAF170, BAF155, BRG1, and BRM [14]. We then analyzed the intersection between the 117 DEGs and the target genes of these core proteins, and we found that there was no statistically significant enrichment (Figure S1A). MCPH1 can also interact directly with E2F1 to maintain genomic stability and chromosome integrity [16]. To investigate whether *Mcph1* affects neural development by E2F1-mediated transcriptional regulation, we analyzed the intersection between the E2F1 target genes (12,834) and 117 DEGs after *Mcph1* deletion. The results showed 51 intersecting genes, accounting for only 44 percent of the DEGs, with at least one E2F1 binding sequence (Figure 3A,B, Table S1). We further performed the biological function analysis for intersecting genes and found them to be mainly enriched in metabolic processes (Figure 3C). Then, based on the number of E2F1-binding sequences, *p* value, fold change, and association with neurodevelopment and disease, we selected 13 genes for verification (Table S1), namely, *Aldoa*, *Crlf2*, *Slc16a3*, *Eif2s3y*, *Eno1*, *Satb2*, *Fn1*, *Hivep2*, *Ldha*, *Cdkn1c*, *Pkm*, *Rabgap1l*, and *Vegfa*. The results showed that *Satb2* (encoded for special AT-rich sequence-binding protein 2) was significantly downregulated, while *Cdkn1c* (encoded for p57), *Crlf2* (encoded for TSLP receptor), and *Slc16a3* (encoded for MCT4) were significantly upregulated (Figure 3D,E and Figure S2). All these data suggest it is unlikely that MCPH1 plays a role as a transcription regulator, especially through interaction with E2F1, to control brain development. To further study whether MCPH1 functions directly to regulate gene expression, for example, *Cdkn1c*, we constructed different length promoter regions of *Cdkn1c* into an expression vector with Firefly luciferase. Through the double luciferase reporting assays, we found that MCPH1 overexpression could enhance rather than repress the transcription activity of the *Cdkn1c* promoter (Figure 3F). However, an increasing expression of *Cdkn1c* was found after the deletion of *Mcph1* (Figure 3D). These results suggest that *Mcph1* does not affect *Cdkn1c* expression through a direct transcriptional regulation function. 

### 2.4. Mcph1 Knockout Blocks Proliferation and Increases Senescence in MEFs

**Figure 4:**
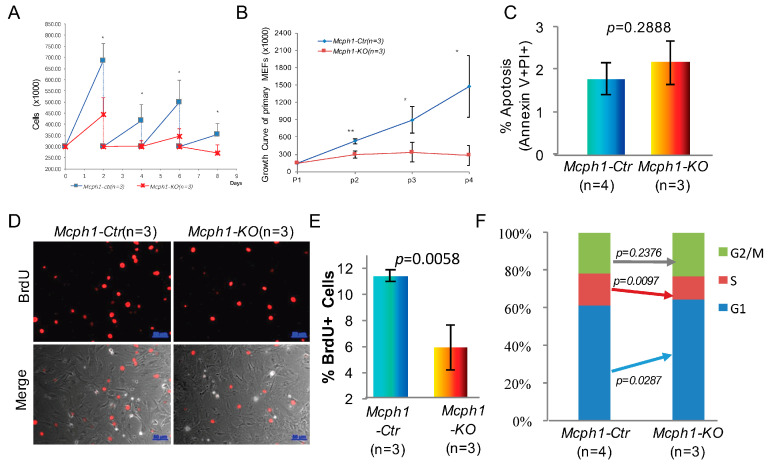

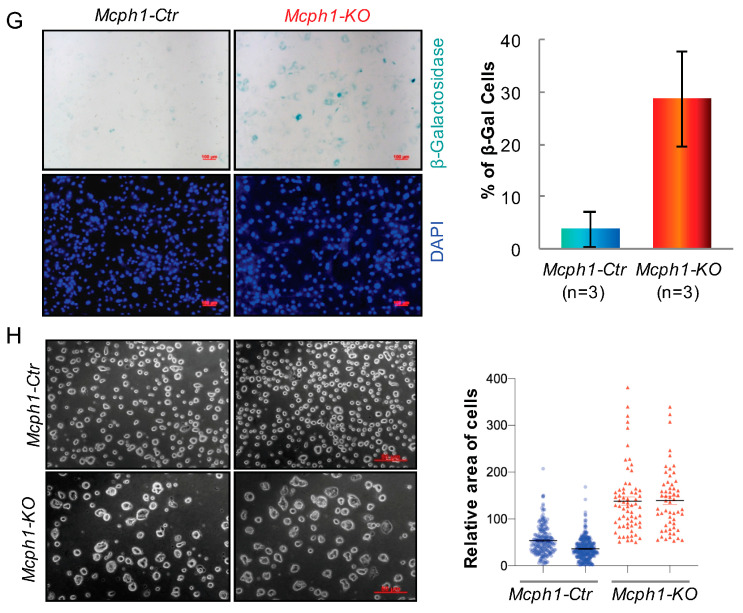
Growth inhibition and senescence in the primary MEFs without *Mcph1*. (**A**) Proliferation analysis of the primary MEFs. The proliferation rate was measured by seeding the same amount (3 × 10^5^) of primary MEFs of indicated genotypes. The cells were passaged every two days, and the cell numbers were determined before passing. The experiment was repeated three times. *, *p* < 0.05. (**B**) The proliferation rate was measured by the number of cells in different passages of indicated genotypes. Bars represent the SEM. Statistical analysis was performed using Student’s *t*-test. *, *p* < 0.05; **, *p* < 0.01. (**C**) Flow cytometry was performed with double Annexin V-FITC/PI staining for *Mcph1-Ctr* and *Mcph1-KO* MEFs. The percentage of apoptosis (% apoptosis) was measured. Bars represent the SEM. A statistical analysis was performed using Student’s *t*-test. (**D**) BrdU labeling of P2 primary MEFs in vitro and incubation for 1 h at 37 °C. The cells were stained with anti-BrdU-antibody (red) to determine the proliferation rate of primary MEFs at P2. Scale bars, 50 µm. (**E**) Quantification of BrdU-positive cells percentage of primary MEFs in *Mcph1-Ctr* and *Mcph1-KO* group. A statistical analysis was performed using Student’s *t*-test. (**F**) Knockout of *Mcph1* disturbed the cell cycle in primary MEFs. The distribution of the cell cycle was detected by flow cytometry with PI staining. The percentages of the G1, S, and G2/M phases were calculated. The representative charts and quantified results of three independent experiments were shown. Statistical analysis was performed using Student’s *t*-test. (**G**) SA-β-gal-positive cells could be observed in the primary MEFs. Cells were subcultured and maintained in a growth medium before assay for β-gal activity staining at early passage. Scale, 100 µm. On the right are the staining levels of SA-β-gal-positive cells, which were analyzed digitally by Image J v1.53 k. The percentage of senescence (% β-Galactosidase cells) was measured. Bars represent the SEM. Statistical analysis was performed using Student’s *t*-test. (**H**) The morphology of control cells and *Mcph1-KO* cells were examined by phase-contrast microscopy. The P4 primary MEFs were trypsinized by trypsin. Scale bar, 50 µm. The figure on the right shows the quantification of the relative area of cells after trypsinization in control and *Mcph1-KO*. Bars represent the SEM.

p57(encoded by *Cdkn1c*) plays a role in regulating the activity of cyclin/CDK complexes, which can cause cell cycle arrest in G1 and inhibit cell proliferation. Since we found that *Mcph1-KO* mice already had slowed growth in the embryonic period, we then isolated primary mouse embryonic fibroblasts (MEFs) from E13.5 embryos of the *Mcph1-Ctr* and *Mcph1-KO* genotypes to investigate the effect of *Mcph1* depletion on cell proliferation. Initially, 3 × 10^5^ cells of the desired genotypes were seeded; then, the cells were trypsinized, counted every two days, and reseeded again at the same number. As shown in Figure 4A, the *Mcph1-KO* MEFs proliferated much more slowly than the control cells. To further test the proliferative ability, 1.5 × 10^5^ cells of the desired genotypes were seeded, then trypsinized, stained with trypan blue, and counted at the indicated time points. The direct cell counts showed that the MEFs in the control group grew faster than those in the *Mcph1-KO* group (Figure 4B). These results demonstrate that the knockout of *Mcph1* can cause delayed growth in primary MEFs. 

To investigate why the deletion of *Mcph1* leads to delayed growth, we isolated primary MEFs from the indicated groups and used flow cytometry to investigate cell apoptosis. As shown in Figure 4C, the disruption of *Mcph1* in the primary MEFs did not increase the percentages of apoptotic cells. Next, the primary MEFs (passage 2, P2) were treated with 10 µM of BrdU for 1 h. The BrdU staining assay showed that more than 10% of the cells were BrdU+ in the control group, while the positive cells in the *Mcph1-KO* group were less than 6% (Figure 4D,E). To further study the proliferation defects, the cell cycle of the primary MEFs was then examined. The percentage of cells in the S phase was significantly lower in the *Mcph1-KO* than in the control group, whereas the number of cells arrested in the G1 phase increased (Figure 4F). These results suggest that the cell growth inhibition caused by the knockout of *Mcph1* is not due to cell apoptosis but to the blockage of cells in the G1/G0 after the *Mcph1* knockout.

Since G1/G0 arrest could lead to cellular senescence, we monitored SA-*β*-gal activity, a well-known senescence marker, in the control and *Mcph1-KO* cells. The percentage of SA-*β*-gal-positive cells in the control group was lower than 10% (Figure 4G). In comparison, about 30% of the *Mcph1-KO* group exhibited positive SA-*β*-gal, indicating that as *Mcph1* is depleted, cell senescence becomes more serious (Figure 4G). Moreover, cells undergoing senescence show significant changes in morphological characteristics. Senescent cells are enlarged and characterized by large nuclei [17]. In order to examine the size of the primary MEFs, we collected P4 MEFs after trypsinization and found an enlarged cell body in the *Mcph1-KO* cells (Figure 4H). These results suggest that the knockout of *Mcph1* can lead to senescence in primary MEFs.

### 2.5. Growth Inhibition in the Mcph1-KO Cells Can Be Rescued by the Depletion of p19Arf

**Figure 5:**
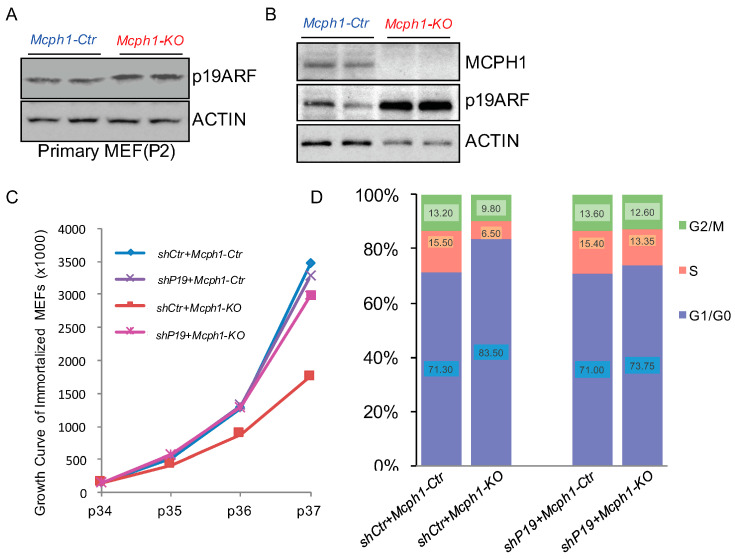
Knockdown of *p19Arf* can rescue *Mcph1-KO* cell growth inhibition. (**A**) Protein lysates were extracted from *Mcph1-Ctr* and *Mcph1-KO* P2 primary MEFs. The p19ARF was examined by Western blotting using an anti-p19ARF antibody. β-ACTIN was used as a loading control. (**B**) Protein lysates were extracted from *Mcph1-Ctr* and *Mcph1-KO* immortalized cells. The MCPH1 protein and p19ARF were examined by Western blotting using an anti-MCPH1 antibody and an anti-p19ARF antibody, respectively. β-ACTIN was used as a loading control. (**C**) The proliferation rate of immortalized cells was measured by the number of cells in different passages of indicated genotypes. *shP19* indicates that *p19Arf* was knocked down in immortalized cells. (**D**) Immortalized cells harvested were fixed and stained with propidium iodide, and their DNA contents were analyzed by flow cytometry. *Mcph1-KO* arrested the cells in the G1/G0 phase, and depletion of *p19Arf* rescued them. Each phase was calculated using the cell ModFit LT v3.2 The percentages of cells in G1, S, and G2/M were also shown as indicated.

MCPH1 has been reported to interact with E2F1 and repress the expression of p14ARF [15], a homolog of mouse p19ARF, both of which are markers of cellular senescence [18]. Indeed, we observed a significant increase in p19ARF in the *Mcph1-KO* groups in the primary MEFs (Figure 5A). However, primary MEFs are commonly grown in cell culture and enter senescence after a low number of passages [19,20]. Senescence is an irreversible stagnation of growth. To avoid the effects of physiological senility, we immortalized the MEFs with the NIH-3T3 protocol. As shown in Figure S3A, when the immortalized cells propagated to P28, they maintained the growth trend, indicating that the immortalized cell line was successfully constructed. Interestingly, the protein expression of p19ARF was also significantly increased after the knockout of *Mcph1* in the immortalized cells (Figure 5B).

Consistent with the results of primary cells, the growth speed of the immortalized *Mcph1-KO* cells was slower than the control group (Figure S3A and Figure 5C). In addition, the BrdU labeling results showed that the number of BrdU+ cells was lower after the *Mcph1* knockout, indicating that the *Mcph1* knockout affected cell proliferation (Figure S3B). Similarly, most cell lines without *Mcph1* stagnated in the G1/G0 phase (Figure S3C). The above data suggest that *Mcph1* knockout does affect cell proliferation and cell cycle.

To study whether cell proliferation defects and cell cycle arrest after *Mcph1* deletion were caused by p19ARF expression in the immortalized MEFs, we knocked down *p19Arf*. Surprisingly, the further knockdown of *p19Arf* in the *Mcph1-KO* cells restored the cell growth to near level of the control group (Figure 5C). Meanwhile, the knockdown of *p19Arf* did not affect cell growth in the control cells (Figure 5C). Furthermore, after the knockdown of *p19Arf*, the number of cells in the G1/G0 phase in the *Mcph1-KO* cells decreased from 83.50% to 73.75%, and the number of cells in the S and M phases also increased (Figure 5D). All these results suggest that silencing *p19Arf* can restore cell proliferation defects and cell cycle arrest caused by *Mcph1* knockout. 

## 3. Discussion

Primary microcephaly is a neurodevelopmental disorder characterized by significantly reduced brain volume without other malformations or significant neurologic defects [9]. In addition, postnatal short stature has been described in two children born to consanguineous parents [8]. Short stature phenotype was also present in some laboratory-constructed mouse models [11]. As mentioned above, our previous study noted a short-stature phenotype in newborn mice [12]. Here, we observed that *Mcph1-KO* animals were smaller during early embryonic development at E14.5 and even earlier at E11.5 (Figure 1). This indicates that the delayed growth phenotype could be present in the early embryonic stage in MPCH. Additionally, *Mpch1* knockout MEFs from a mouse model generated by Liu lacking the N′-BRCT domain of MCPH1 were reported to proliferate more slowly than control cells [21]. Our results also indicated that both the primary and immortalized *Mcph1* knockout MEFs showed cellular proliferation defects (Figure 4A,B and Figure 5C). Thus, the knockout of *Mcph1* impairs cell growth.

MCPH1 binds to E2F1 and plays a role in transcriptional regulation. E2F1 is a multifunctional transcription factor with a large number of target genes, which can participate in cell cycle, DNA damage response, cell apoptosis, and cell metabolism [22,23,24,25]. Microcephaly is also commonly seen in DNA damage repair-related gene function loss and cell cycle regulation dysfunction [26,27,28]. MCPH1 is involved in cell cycle regulation, and MCPH1 patient cells exhibit delayed release from DNA damage-induced G2/M checkpoint arrest [28]. However, as few as 117 DEGs were found after the knockout of *Mcph1*, and only half of them were upregulated (Figure 2D), which does not match the suggested transcriptional repression function of MCPH1. Secondly, only 51 (out of 117) DEGs intersected with the E2F1 target genes, and the intersection was not statistically significant (Figure 3A). Thirdly, the expression of DEGs was predicted to be regulated mainly by transcription factors SP7, EP300, HES1, NR4A2, SMAD3, ETS1, CTNNB1, HIF1A, SMAD1, and NFKB1 rather than E2F1 (Figure S1B). Fourthly, although the E2F1 target gene *Cdkn1c* was upregulated in the *Mcph1-KO* brain, the overexpression of MCPH1 enhances, rather than represses, the promoter activity of *Cdkn1c* (Figure 3F). Therefore, *Mcph1* is unlikely to affect neurodevelopment through E2F1-mediated transcriptional activity.

Interestingly, RNA-seq data analysis showed that *Mcph1* mainly affected metabolic processes, followed by developmental processes, which is consistent with previous studies [29]. Nathalie Journiac et al. have shown that the knockout of *Mcph1* significantly affects cellular metabolism, as well as the cell cycle [29] (see also Figure S4A). We performed the same analysis of our DEGs and found that they were co-enriched in metabolism and development (Figure S4B). Cyclins can regulate glucose uptake and utilization through direct and indirect pathways [30]. Due to ATP requirements, cells rely primarily on TCA cycling during the G1 phase while switching to glycolysis during the S phase, where glucose metabolism may be regulated in a cell cycle-dependent manner [31]. These suggest that *Mcph1* may cause cell cycle-dependent metabolic changes.

Cell cycle-related cell proliferation is negatively regulated by the Cip/Kip and INK4 families [32]. The Cip/Kip family (including *CDKN1A*, *CDKN1B*, and *CDKN1C*) and the INK4 family (including *p16INK4a*, *p15INK4b*, *p18INK4c*, and *p19INK4d*) regulate the activity of the cyclin/CDK complexes to arrest cell cycle in the G1 phase and inhibit cell growth [33]. p16INK4a and p14ARF (p19ARF in mouse) are encoded by *Cdkn2a* [34]. In early embryonic development, p57 (encoded by *Cdkn1c*) is highly expressed [35]. In contrast, p19ARF (encoded by *Cdkn2a*) is not expressed until E17.5 in the embryo and is highly expressed mainly in the testis and spleen after birth [36]. However, the expression of p19ARF was observed in MEFs isolated from E13.5 embryos [36]. It has been reported that p57 binds to E2F1 and negatively regulates RNA polymerase II C-terminal domain phosphorylation in an E2F1-dependent manner [37]. In addition, loss-of-function mutations in *CDKN1C* can cause microcephaly [38]. Our data show that *Mcph1* knockout caused a significant upregulation of *Cdkn1c* in the brain (Figure 3D). Increased *Cdkn1c* expression may prolong the G1 phase progression, stop the proliferation of stem cells, and decrease the number of cells, finally leading to small body size and the abnormal development of mice (Figure 1). 

Human MCPH1 has been reported to repress the expression of cellular senescence marker p14ARF [18]. Indeed, in the *Mcph1* knockout MEFs, p19ARF, a homolog of human p14ARF, was significantly increased (Figure 5A). Moreover, when *Mcph1* was depleted in the MEFs, we observed a distortion of the cell cycle with an enhanced portion of the G1 phase and decreasing S phase cells, reduction in BrdU-positive cells, and increase in the number of senescent cells (Figure 4, Figure 5 and Figure S3). It was reported that *p19ARF*-null MEFs grown in culture have a higher proliferative capability than MEFs that are wild-type for p19ARF [39]. Consistent with this, when we silenced *p19Arf* in the *Mcph1* knockout cell line, we observed that the growth curve returned to normal, and at the same time, the cell cycle returned to normal (Figure 5C,D), suggesting that delayed growth in *Mcph1-KO* can be rescued by the depletion of *p19Arf*. All these indicated that *Mcph1* may control cell cycle and cellular senescence through regulating *p19Arf.*

So, what causes the increase in *Cdkn1c* and p19ARF, leading to cell cycle arrest and cell senescence after *Mcph1* depletion? *Mcph1* is a well-known function in DNA damage response and repair. MCPH1 can be recruited into DNA double-strand breaks, and it promotes DDR by binding to SWI/SNF chromatin remodeling agents [14]. The BRUCE-USP8-BRIT1 complex promotes chromatin relaxation, allowing downstream DNA damage signaling and repair proteins to enter [40]. Our previous research proposed a possible link between DNA repair and microcephaly [12]. Liu pointed out that DDR signaling and DNA repair were impaired in *Mcph1-ΔBR1* primary MEFs [21]. In MEFs, DNA damage caused by exposure to γ-radiation can increase the expression of p19ARF, thereby causing cell cycle arrest [41]. Although we have not further investigated the direct relationship between DDR and p19ARF expression in *Mcph1* knockout cells, it is reasonable to speculate that the knockout of *Mcph1* may increase p19ARF by causing DNA damage and then lead to cell cycle arrest and senescence.

## 4. Materials and Methods

### 4.1. Mice

One *Mcph1*^+\−^ male mouse and two *Mcph1*^+\−^ female mice were combined in the cage, and the mice were separated after checking the vaginal plug [5]. After 12 days and 14 days of gestation, the female mice were killed. The embryos were removed and photographed. PCR determined the genotype analysis of the *Mcph1* knockout (designated as *Mcph1-KO*) mice on DNA extracted from tail tissue. The mice were kept in a specific pathogen-free Sun Yat-sen University (SYSU) animal facility, and the experiments were conducted according to the SYSU Institutional Animal Care and Use Committee (SYSU IACUC). The mice were fed standard laboratory food and water in ventilated cages under a 12 h light/dark cycle.

### 4.2. RNA-Seq and Data Analysis

The brains of embryos at E14.5 were isolated, and three from each group (controls and *Mchp1-KO*) were sent to BGI.

Raw readings were preprocessed using standard Illumina pipelines to separate the multiplexed readings. Using the FastQC program, we checked the sequence quality. The data were compared to the mouse reference genome (mm10 version) using HISAT2 v2.1.0. The results were sorted using SAMtools v1.9 comparisons, and all fragment quantifications were calculated using HTSeq v0.11.2. Finally, edgR v3.15 was used for differential expression analysis. We then obtained the differentially expressed genes under the two conditions. In total, 117 genes were identified as strictly differentially expressed genes with a fold change greater than 1.2 and adjusted *p*-values of less than 0.05.

We then used Metascape for GO enrichment and KEGG pathway analysis [42]. First, all the DEGs were analyzed. Then, the DEGs were divided into upregulated genes and downregulated genes, and they were analyzed separately. The results of the downregulated gene analysis were not statistically significant. 

We used the GTRD database to find the target genes of E2F1 and the target genes of BAF155, BAF170, BRG1, and BRM, the core proteins in the SWI/SNF complex [43]. Then, we analyzed the intersecting genes between the E2F1 target genes and the *Mcph1-KO* DEGs. The intersection was based on at least one E2F1-binding sequence. We used Enrichr to perform transcription factor prediction analysis of the *Mcph1-KO* DEGs [44,45,46]. 

### 4.3. RNA Isolation and PCR Analysis

For RNA extraction, 1 mL trizol reagent (15596026, Thermo Scientific, Waltham, MA, USA) was added to the cells in a 6-well plate, and 0.2 mL chloroform (87070P, Adamas, Shanghai, China) was added after oscillation. We collected the lysate and centrifuged; then, the upper colorless aqueous phase was taken, and 0.5 mL isopropanol (GHTECH, Guangzhou, China) was added. After centrifugation, the precipitation was collected and washed with 1 mL of 75% ethanol (GHTECH, Guangzhou, China). We dried the pellet and dissolved it in RNA-free water (P071-01, vazyme, Nanjing, China).

The above RNA was used to synthesize the first-strand cDNA using the RevertAid First Strand cDNA Synthesis Kit (K1622, Thermo Scientific, Waltham, MA, USA), according to the manufacturer’s instructions. SYBR Green Premix (AG11701, Accurate, Changsha, China) was used for the quantitative PCR (qPCR) reactions. Table S2 shows the list of primers used in this study.

### 4.4. Primary MEFs Isolation and Culture

Primary mouse embryonic fibroblasts were isolated from E13.5 embryos [9], and the fetal livers were collected for DNA isolation and PCR-genotyping to determine the genotype of the embryos. The embryos were transferred into a cryovial containing ice-cold PBS (BL302A., Biosharp, Beijing, China) and cut into small pieces. The minced tissue was digested with trypsin-EDTA-solution (25300, Life Technologies, Carlsbad, CA, USA) for 4 min, and then the supernatant was transferred to tubes containing MEF medium. The primary MEFs were cultured at 37 °C with 5% CO_2_.

### 4.5. Proliferation Assay of Primary MEFs

The primary MEFs of the specified genotypes were plated into one well of a 6-well plate with a 3 × 10^5^/well density. The number of cells was counted and passed every three days, and the same number of cells was inoculated each time.

### 4.6. BrdU Labeling and Staining

The cells were seeded onto slides and cultured at 37 °C and 5% CO_2_. After incubation for 24 h, BrdU (final concentration 10 µM, QIA58, Sigma, St. Louis, MO, USA) was added to the medium and incubated for 1 h. The cells were fixed with 4% paraformaldehyde (BL539, Biosharp, Beijing, China), treated with 2N HCl at 37 °C for 30 min, sealed, and made permeable with blocking solution (BS) (5% goat serum (SL038, Solarbio, Beijing, China), 1% bovine serum albumin (A7030, Sigma, St. Louis, MO, USA), 0.4% Triton X-100 (T8787, Sigma, St. Louis, MO, USA)). BrdU antibody (1:200 dilution, ab8152, Abcam, Cambridge, UK) was incubated overnight at 4 °C, and then the secondary antibody Alexa Fluor 555 (1:200, A31570, Invitrogen, Carlsbad, CA, USA) was incubated at room temperature for 2 h. A mounting agent (F4680, Sigma, St. Louis, MO, USA) was used to seal the plates, and the images were observed under a fluorescence microscope (ZEISS, Axio Observer 7, Oberkochen, Germany). Each experiment was conducted at least 3 times, and the results were shown as the percentage of BrdU-positive cells in the histogram.

### 4.7. Cell Cycle Analysis

The primary MEFs, both control and *Mcph1-KO*, were seeded in 60 mm dishes at a 1 × 10^6^ cells/dish density. After incubation for 72 h, the cells were harvested, centrifuged to remove the supernatant, and washed twice with PBS. The cells were fixed in cold 70% ethanol for at least 24 h (4 °C) and then centrifuged at 200× *g* (room temperature) for 5 min. Then, the cells were washed twice with FACS buffer and treated with 200 µg/mL RNase A (R6513, Sigma, St. Louis, MO, USA) for 1 h (37 °C), and then stained with 40 µg/mL propidium iodide (PI) solution (537060, Calbiochem, San Diego, CA, USA) at room temperature. After 30 min of DNA labeling, they were transferred to a flow cytometry tube for cell cycle analysis in a BD LSR Fortessa flow cytometer (BD Biosciences, San Jose, CA, USA). Finally, FACS Diva v6.3 was used for cell cycle analysis.

### 4.8. Annexin Apoptosis Assays

For primary MEFs control and *Mcph1-KO*, 1 × 10^6^ cells were plated on 60 mm dishes for 48 h. After that, the cells were treated with 7.5 µM of nocodazole (M1404, Sigma, St. Louis, MO, USA) for 24 h. Subsequently, the cells were harvested, washed with FACS, and stained with the commercial kit Annexin-V-FITC apoptosis detection kit (C10625, Beyotime, Shanghai, China) to analyze live, early, and late apoptotic and necrotic cells by flow cytometry using a BD LSR Fortessa flow cytometer (BD Biosciences, San Jose, CA, USA).

### 4.9. Senescence-Associated β-Galactosidase Staining

A proper number of cells were seeded into multiple wells in a 12-well plate and cultivated for 24 h. Then, according to the manual, the Senescence Cells Histochemical Staining Kit (CS0030, Sigma, St. Louis, MO, USA) was used to perform SA-β-Gal staining on different groups of primary MEFs. Photos were taken under an inverted microscope.

### 4.10. Western Blotting

Tissues or cells were lysed with RIPA buffer (50 mM Tris-HCl (DB, Shanghai, China) pH 7.4, 150 mM NaCl (DB, Shanghai, China), 1% NP40 (I8896, Sigma, St. Louis, MO, USA), 1 mM EDTA (DB, Shanghai, China), plus 1 tablet of Roche complete protease inhibitor (11836170001, Roche, Basel, Switzerland) per 10 mL) to collect total protein. Then, the total protein was examined using 12% SDS-PAGE gel electrophoresis. The proteins on the gel were then transferred to the PVDF membrane (1620177, Bio-Rad, Hercules, CA, USA), which was blocked with 5% milk (FD6006, FUDE, Hangzhou, China) for 1 h. Primary antibody MCPH1 (1:2000, 4120, Cell Signaling, Danvers, MA, USA), p19ARF (ab80, Abcam, Cambridge, UK), and β-ACTIN (1:5000, A5441, Sigma, St. Louis, MO, USA) were incubated overnight at 4 °C. We incubated the secondary antibody with HRP for 1 h at room temperature. Signals were visualized with the Super Signal Chemiluminescent Substrate (32106, Thermo Scientific Waltham, MA, USA).

### 4.11. Luciferase Reporter Assay

Fragments of *Cdkn1c* with different promoter lengths were constructed and inserted into the PGL3 plasmid. P-C-1000 indicates that it starts at 1000 bp after the start codon, and the promoter length is about 900 bp; P-C-2000 indicates that it starts at 1000 bp after the start codon, and the promoter length is about 350 bp. The promoter experiments were performed with N2a cells in a 24-well format. We used 300 ng pGL3-Cdkn1c-1000/pGL3-Cdkn1c-2000, pGL3-Basic; 600 ng pCDNA3.0-HA, or pCDNA3.0-HA-Mcph1; 18.75 ng pGL4.74 vector was used for the cotransfection of cells. Then, the cells were lysed, and luciferase activities were determined using the Dual-Luciferase ® Reporter Assay System (E1910, Promega, Madison, WI, USA) and GloMax^®^ Discover System (Promega, Madison, WI, USA).

## 5. Conclusions

In summary, we found that delayed neurodevelopment in *Mcph1* knockout mice is unlikely to be regulated by E2F1-mediated transcription repression function. The cell cycle arrest and senescence caused by *Mcph1* knockout may be due to DNA damage accumulation, which leads to the increasing expression of key cell cycle regulators such as *Cdkn1c* (encoded for p57) or p19ARF. Thus, we concluded that *Mcph1* prevents neurodevelopment abnormality and delayed growth through cell cycle regulation independent of its transcriptional function. 

## Data Availability

The data presented in this study are available on request from the corresponding authors.

## Supplementary Materials

The supporting information can be downloaded at: https://www.mdpi.com/article/10.3390/ijms25094597/s1. References [29,47,48,49,50,51,52,53,54,55,56,57,58,59] are cited in the supplementary materials.
