# Supplementary material for: Microcephaly Gene Mcph1 Deficiency Induces p19ARF-Dependent Cell Cycle Arrest and Senescence
Source: Int J Mol Sci. 2024 Apr 23;25(9):4597. doi: 10.3390/ijms25094597 (PMC11083351; doi:10.3390/ijms25094597)
Supplement: Supplementary file 1 [file ijms-25-04597-s001.zip › ijms-2935204-supplementary.pdf]

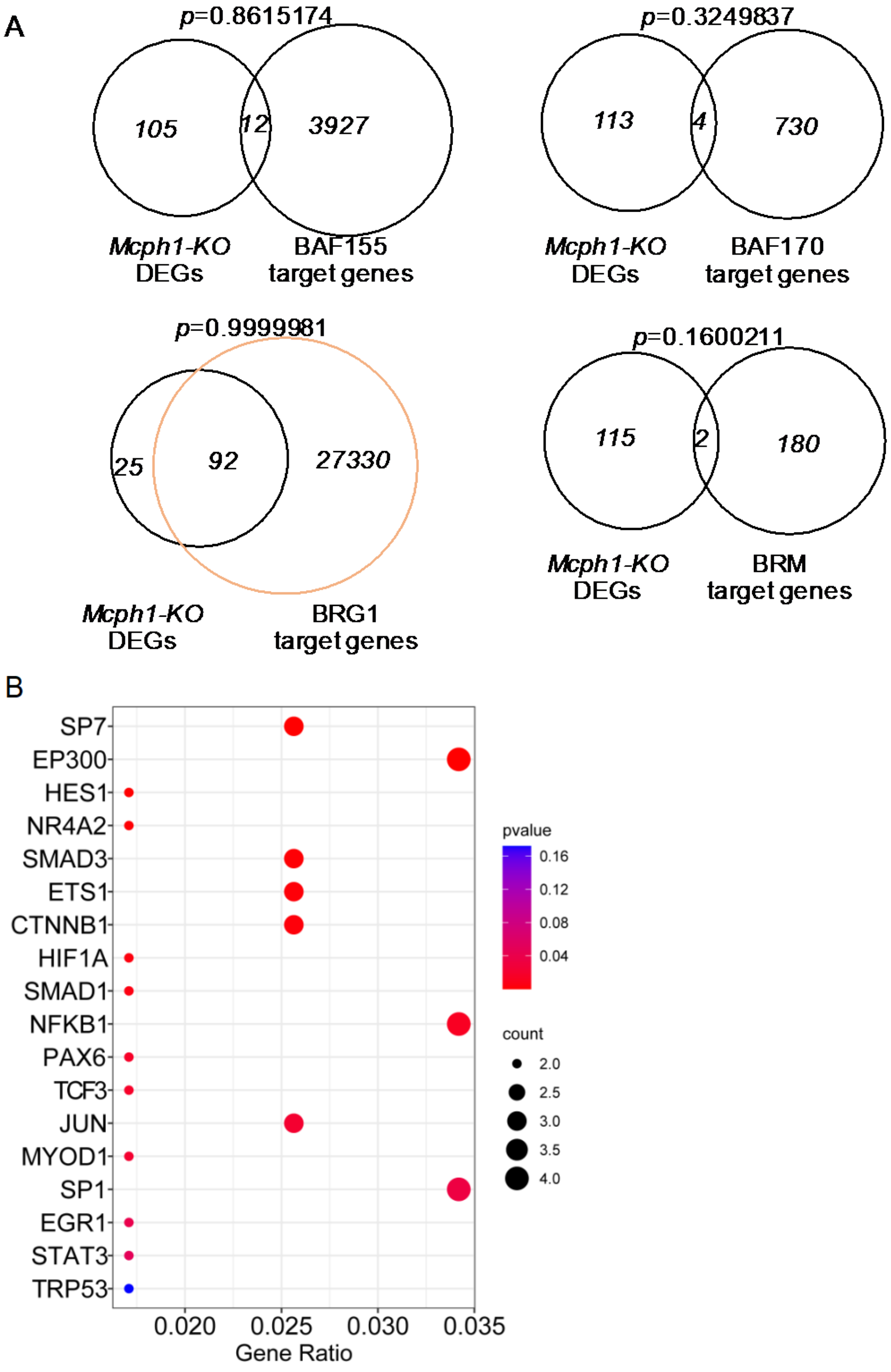


**Figure S1.** *Mcph1* DEGS were not associated with transcriptional regulation of the SWI/SNF complex and E2F1. (A) The Venn diagrams represent the number of intersecting genes between *Mcph1-KO* DEGs and the target genes of the core proteins of the SWI/SNF complex. The core proteins in this complex are BAF155, BAF170, BRG1, and BRM. $p > 0.05$ indicates no statistical significance. Statistical analysis was performed using Fisher's exact test. (B) *Mcph1-KO* DEGs transcription factor prediction analysis.

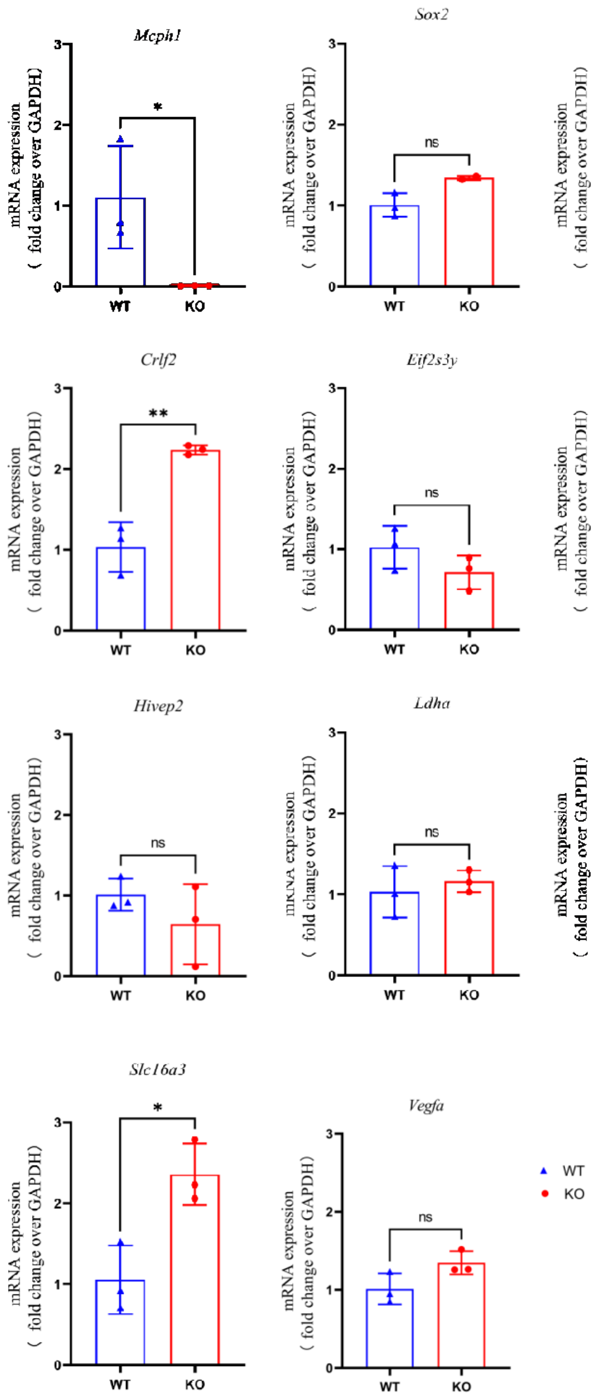


**Figure S2.** Expression verification of intersecting genes related to neurodevelopment. WT is the control group, and KO is the knockout of the *Mcph1* group. The expression of DEGs in the control and *Mcph1-KO* groups was detected by RT-qPCR.

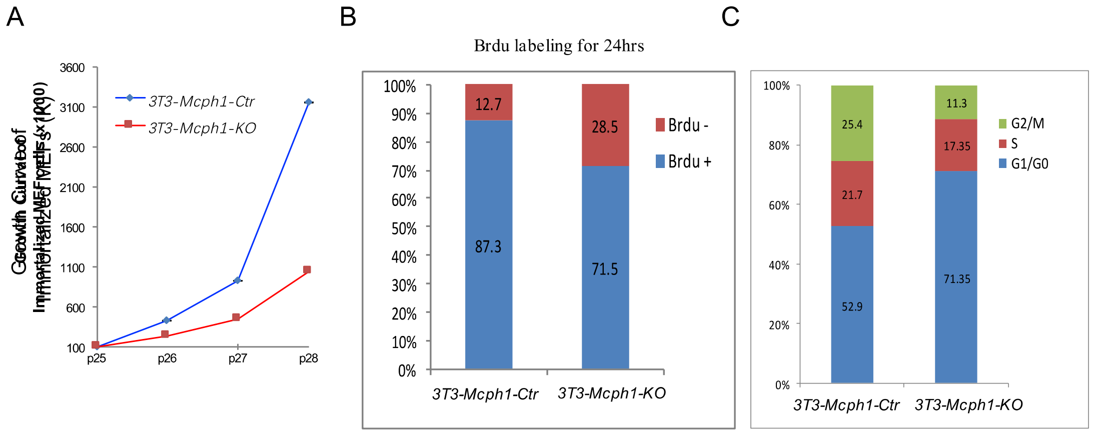


**Figure S3.** *Mcph1* affects cell proliferation in immortalized MEFs. (A) Growth curves of *Mcph1-Ctr* and *Mcph1-KO* immortalized MEFs. The immortalized cells have a good proliferation capability. Cells were harvested by trypsinization and counted once per passage. P25, cells at passage 25; P26, cells at passage 26; P27, cells at passage 27, and P28, cells at passage 28. (B) Immobilized MEFs, stained with BrdU antibody, and quantitative flow cytometric analysis showed that BrdU-labeled *Mcph1-KO* cells decreased at 24 h. (C) The distribution of the cell cycle was detected by flow cytometry with PI staining. The percentages of the G1, S, and G2/M phases were calculated.

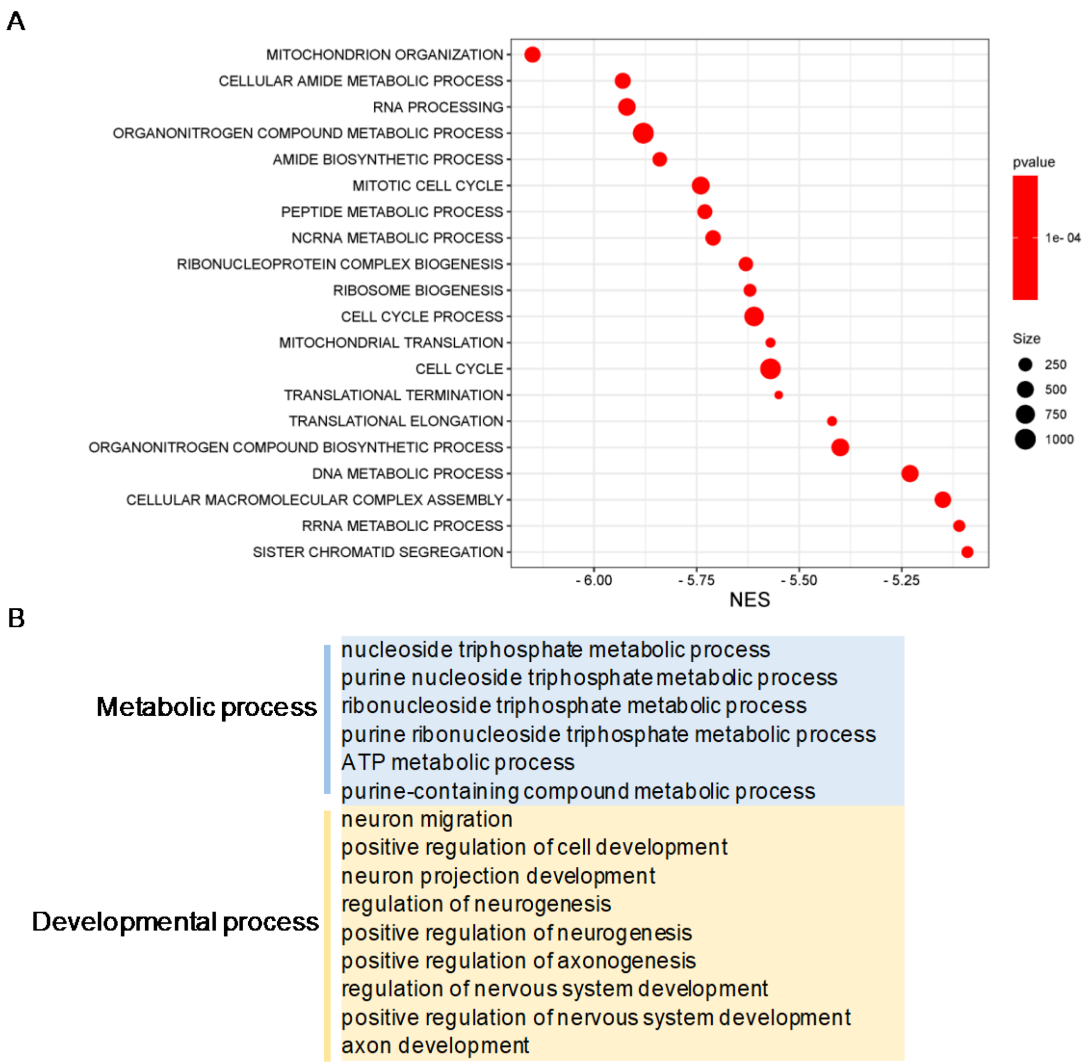


**Figure S4.** *Mcph1* may cause cell cycle-dependent metabolic changes (A) The biological process of the DEGs in the results of Nathalie Journiac et al[1]. The figure shows the top 20 biological processes, including metabolism-related and cell cycle related (B) We used metascape to perform the same analysis on our DEGs and their DEGs[1]. The intersection of the two different genes focuses on metabolism and development.

**Table S1.** Summary of intersecting genes information

| NO. | Intersecting Gene | log2FoldChange | *P* adj | Site Count* | Remark |
|---|---|---|---|---|---|
| 1 | *Ldha* | 0.53258291 | 1.40E-06 | 4 | *Ldha* can affect neuronal excitability[2]. |
| 2 | *Tcf7l1* | 0.534401708 | 0.047987897 | 4 | - |
| 3 | *Vegfa* | 0.584031025 | 0.00033305 | 3 | *Vegfa* affects embryonic angiogenesis[3]. |
| 4 | *Hey1* | 0.460385188 | 0.029574475 | 3 | - |
| 5 | *Aldoa* | 0.310349563 | 0.047987897 | 3 | *Aldoa* is related to cytoskeletal development and function[4]. |
| 6 | *Slc16a3* | 1.274481753 | 9.16E-06 | 2 | Deletion of *Slc16a3* is likely to result in decreased lactate in the embryo's blood and a decrease in the embryo's growth[5, 6]. |
| 7 | *Tpi1* | 0.444301423 | 8.25E-05 | 2 | - |
| 8 | *Eno1* | 0.436598496 | 0.000176167 | 2 | *Eno1* inactivation causes delayed brain development[7, 8]. |
| 9 | *Crlf2* | 0.884397108 | 0.000936195 | 2 | Crlf2 is involved in neuronal signaling, and patients have symptoms of microcephaly[9]. |
| 10 | *Fn1* | 0.378091826 | 0.001353569 | 2 | Mouse embryos lacking *Fn1* have defects in mesoderm and neural tube development[10]. |
| 11 | *Lcp1* | 0.521461793 | 0.002515204 | 2 | - |
| 12 | *Pkm* | 0.338713642 | 0.003225653 | 2 | *Pkm* is related to synaptic plasticity[2, 11]. |
| 13 | *Rabgap1l* | -0.342916124 | 0.00422707 | 2 | *Rabgap1l* is differentially methylated in Zika-induced microcephaly[12]. |
| 14 | *Spp1* | 1.198484624 | 0.011059793 | 2 | - |
| 15 | *Hivep2* | -0.348226286 | 0.016053953 | 2 | Mutations in the *Hivep2* gene cause developmental delay/intellectual disability[13]. |
| 16 | *Nxn* | 0.40763114 | 0.01743748 | 2 | - |
| 17 | *Eif2s3y* | -0.42710408 | 0.019913806 | 2 | *Eif2s3y* can inhibit the pluripotency state of embryonic stem cells in mice[8]. |
| 18 | *Gm37844* | -0.88957664 | 0.021387194 | 2 | - |
| 19 | *Inhba* | -0.8904405 | 0.046788412 | 2 | - |
| 20 | *Ddit4* | 0.469127086 | 0.047987897 | 2 | - |
| 21 | *Satb2* | -0.72840142 | 2.19E-06 | 1 | Mutations in the *Satb2* gene cause developmental delay/intellectual disability[6]. |
| 22 | *Sla* | -0.603553268 | 0.000122916 | 1 | - |
| 23 | *9130024F11 Rik* | -0.710580795 | 0.00033305 | 1 | - |

**Table S1.**（*Continued*）

| NO. | Intersecting Gene | log2FoldChange | *P* adj | Site Count* | Remark |
|---|---|---|---|---|---|
| 24 | *Mef2c* | -0.414368576 | 0.001570872 | 1 | - |
| 25 | *Fam49a* | -0.342475004 | 0.002471334 | 1 | - |
| 26 | *Alas2* | -1.196964327 | 0.002515204 | 1 | - |
| 27 | *Crb2* | 0.509924247 | 0.002920479 | 1 | - |
| 28 | *Pfkl* | 0.373654061 | 0.003110287 | 1 | - |
| 29 | *Dlk1* | 0.556029226 | 0.003225653 | 1 | - |
| 30 | *2610318N02 Rik* | 0.754241912 | 0.003260079 | 1 | - |
| 31 | *Dok6* | -0.398228958 | 0.004321018 | 1 | - |
| 32 | *Pgk1* | 0.352225734 | 0.006154432 | 1 | - |
| 33 | *Gnai1* | -0.359240218 | 0.007113982 | 1 | - |
| 34 | *Dll3* | 0.612639822 | 0.009379012 | 1 | - |
| 35 | *Cdkn1c* | 0.335603094 | 0.011059793 | 1 | Mutations in the *Cdkn1c* gene cause developmental delay/intellectual disability[14]. |
| 36 | *Fut10* | -0.926136173 | 0.011932979 | 1 | - |
| 37 | *Neurog2* | 0.401683379 | 0.011932979 | 1 | - |
| 38 | *Ccnd3* | 0.44717396 | 0.019519963 | 1 | - |
| 39 | *1700048O20 Rik* | 0.804420254 | 0.019519963 | 1 | - |
| 40 | *Slc2a1* | 0.399721042 | 0.030723065 | 1 | - |
| 41 | *Nr4a3* | -0.425268553 | 0.030892622 | 1 | - |
| 42 | *Kcnn1* | 0.55633904 | 0.031913054 | 1 | - |
| 43 | *Gria2* | -0.279563023 | 0.032771223 | 1 | - |
| 44 | *Tmem132b* | -0.320917809 | 0.032952499 | 1 | - |
| 45 | *Mctp1* | -0.698451514 | 0.03545619 | 1 | - |
| 46 | *Neurog1* | 0.770946401 | 0.040967524 | 1 | - |
| 47 | *Rhbdl3* | 0.390346143 | 0.043599623 | 1 | - |
| 48 | *Necab1* | -0.486480137 | 0.046488149 | 1 | - |
| 49 | *Mpped1* | -0.309551635 | 0.047922476 | 1 | - |
| 50 | *Ntrk3* | -0.277612814 | 0.047987897 | 1 | - |
| 51 | *Zic3* | 0.368405971 | 0.049918514 | 1 | - |

* Count of E2F1 binding sequence

**Table S2.** Primers used in this study

| Gene name | Primer | Sequence (5'to 3') |
|---|---|---|
| *Satb2* | Forward Primer | GAGATGAGTTGAAGAGGGCTAGTG |
| | Reverse Primer | CCCTGTGTGCGGTTGAAT |
| *Ldha* | Forward Primer | AGCGTACCCGTGATGCTAAC |
| | Reverse Primer | CAGGGTTGGCAGATCGACAT |
| *Aldoa* | Forward Primer | AACGGTCACACACTTCGTCG |
| | Reverse Primer | TACTTTCCTTGACAAGCGAGGC |
| *Vegfa* | Forward Primer | GCAGGCTGCTGTAACGATGAA |
| | Reverse Primer | TGCTTTCTCCGCTCTGAACAA |
| *Eif2s3y* | Forward Primer | ATCTTGTCCTCAACCTCAGACT |
| | Reverse Primer | TTCTTTAGCCTGGCTTTCTTTCA |
| *Hivep2* | Forward Primer | CTCCTTTCTCCTCCCGAGCG |
| | Reverse Primer | GATCCCGAGGCTACTGGCTG |
| *Rabgap1l* | Forward Primer | ACTGGGAATCTTCATGAGAAGCTGA |
| | Reverse Primer | TCACATTACTGTGCTTGATACACCA |
| *Pkm* | Forward Primer | GGAGGAGGAATGCAGGACTGG |
| | Reverse Primer | GGAGTGCACAAGAAGTGGGGA |
| *Fn1* | Forward Primer | AACAAGAGACCACTGGCACC |
| | Reverse Primer | AGAGGATTGCTTTCCCTGCC |
| *Eno1* | Forward Primer | CGACTGTATGGAATCCAAGGCA |
| | Reverse Primer | CCAGCTTTGCAGACAGCCA |
| *Crlf2* | Forward Primer | GCAGGTGATGTCACAGTCGT |
| | Reverse Primer | GCGCTGCCTAGCCTTAAACA |
| *Slc16a3* | Forward Primer | GGCTGTTTTATCATCACGGGTT |
| | Reverse Primer | GTGTCGCTGTAGCCAATCCC |
| *Cdkn1c* | Forward Primer | AGCTGAAGGACCAGCCTCTCTC |
| | Reverse Primer | ACGTCGTTCGACGCCTTGTTCT |
| *mGapdh* | Forward Primer | GCACAGTCAAGGCCGAGAAT |
| | Reverse Primer | GCCTTCTCCATGGTGGTGAA |
| 1000-Cdkn1c-pGL3 | Forward Primer | TTACGCGTGCTAGCCCGGGCTCGAGGTTG GAGGGCTAGATGGGGAACTT |
| | Reverse Primer | ACCTTAGTTGGCTGGAAGTAGTTATGCTA GAAAAG |
| 2000-Cdkn1c-pGL3 | Forward Primer | TTACGCGTGCTAGCCCGGGCTCGAGGGGG GTCGAATATGGCCTGA |
| | Reverse Primer | atgcagatcgcagatctcgagCTGCACCAACTGATT AGGGCTT |

**References**


1. Journiac, N.; Gilabert-Juan, J.; Cipriani, S.; Benit, P.; Liu, X.; Jacquier, S.; Faivre, V.; Delahaye-Duriez, A.; Csaba, Z.; Hourcade, T.; Melinte, E.; Lebon, S.; Violle-Poirsier, C.; Oury, J. F.; Adle-Biassette, H.; Wang, Z. Q.; Mani, S.; Rustin, P.; Gressens, P.; Nardelli, J., Cell Metabolic Alterations due to Mcph1 Mutation in Microcephaly. *Cell Rep* **2020,** 31, (2), 107506.
2. Yao, S.; Xu, M. D.; Wang, Y.; Zhao, S. T.; Wang, J.; Chen, G. F.; Chen, W. B.; Liu, J.; Huang, G. B.; Sun, W. J.; Zhang, Y. Y.; Hou, H. L.; Li, L.; Sun, X. D., Astrocytic lactate dehydrogenase A regulates neuronal excitability and depressive-like behaviors through lactate homeostasis in mice. *Nat Commun* **2023,** 14, (1), 729.
3. Casas, B. S.; Vitória, G.; do Costa, M. N.; Madeiro da Costa, R.; Trindade, P.; Maciel, R.; Navarrete, N.; Rehen, S. K.; Palma, V., hiPSC-derived neural stem cells from patients with schizophrenia induce an impaired angiogenesis. *Transl Psychiatry* **2018,** 8, (1), 48.
4. Hoffman, J. L.; Faccidomo, S.; Kim, M.; Taylor, S. M.; Agoglia, A. E.; May, A. M.; Smith, E. N.; Wong, L. C.; Hodge, C. W., Alcohol drinking exacerbates neural and behavioral pathology in the 3xTg-AD mouse model of Alzheimer's disease. *Int Rev Neurobiol* **2019,** 148, 169-230.
5. Moreau, J. L.; Artap, S. T.; Shi, H.; Chapman, G.; Leone, G.; Sparrow, D. B.; Dunwoodie, S. L., Cited2 is required in trophoblasts for correct placental capillary patterning. *Dev Biol* **2014,** 392, (1), 62-79.
6. Zarate, Y. A.; Bosanko, K. A.; Caffrey, A. R.; Bernstein, J. A.; Martin, D. M.; Williams, M. S.; Berry-Kravis, E. M.; Mark, P. R.; Manning, M. A.; Bhambhani, V.; Vargas, M.; Seeley, A. H.; Estrada-Veras, J. I.; van Dooren, M. F.; Schwab, M.; Vanderver, A.; Melis, D.; Alsadah, A.; Sadler, L.; Van Esch, H.; Callewaert, B.; Oostra, A.; Maclean, J.; Dentici, M. L.; Orlando, V.; Lipson, M.; Sparagana, S. P.; Maarup, T. J.; Alsters, S. I.; Brautbar, A.; Kovitch, E.; Naidu, S.; Lees, M.; Smith, D. M.; Turner, L.; Raggio, V.; Spangenberg, L.; Garcia-Miñaúr, S.; Roeder, E. R.; Littlejohn, R. O.; Grange, D.; Pfotenhauer, J.; Jones, M. C.; Balasubramanian, M.; Martinez-Monseny, A.; Blok, L. S.; Gavrilova, R.; Fish, J. L., Mutation update for the SATB2 gene. *Hum Mutat* **2019,** 40, (8), 1013-1029.
7. El Waly, B.; Mignon-Ravix, C.; Cacciagli, P.; Buhler, E.; Ben Zeev, B.; Villard, L., Molecular characterization of a 1p36 chromosomal duplication and in utero interference define ENO1 as a candidate gene for polymicrogyria. *Eur J Hum Genet* **2020,** 28, (12), 1703-1713.
8. Li, N.; Mu, H.; Zheng, L.; Li, B.; Wu, C.; Niu, B.; Shen, Q.; He, X.; Hua, J., EIF2S3Y suppresses the pluripotency state and promotes the proliferation of mouse embryonic stem cells. *Oncotarget* **2016,** 7, (10), 11321-31.
9. Paduano, F.; Colao, E.; Loddo, S.; Orlando, V.; Trapasso, F.; Novelli, A.; Perrotti, N.; Iuliano, R., 7q35 Microdeletion and 15q13.3 and Xp22.33 Microduplications in a Patient with Severe Myoclonic Epilepsy, Microcephaly, Dysmorphisms, Severe Psychomotor Delay and Intellectual Disability. *Genes (Basel)* **2020,** 11, (5).
10. George, E. L.; Georges-Labouesse, E. N.; Patel-King, R. S.; Rayburn, H.; Hynes, R. O., Defects in mesoderm, neural tube and vascular development in mouse embryos lacking fibronectin. *Development* **1993,** 119, (4), 1079-91.
11. Ferguson, L.; Hu, J.; Cai, D.; Chen, S.; Dunn, T. W.; Pearce, K.; Glanzman, D. L.;

Schacher, S.; Sossin, W. S., Isoform Specificity of PKMs during Long-Term Facilitation in Aplysia Is Mediated through Stabilization by KIBRA. *J Neurosci* **2019,** 39, (44), 8632-8644.

12. Anderson, D.; Neri, J.; Souza, C. R. M.; Valverde, J. G.; De Araújo, J. M. G.; Nascimento, M.; Branco, R. C. C.; Arrais, N. M. R.; Lassmann, T.; Blackwell, J. M.; Jeronimo, S. M. B., Zika Virus Changes Methylation of Genes Involved in Immune Response and Neural Development in Brazilian Babies Born With Congenital Microcephaly. *J Infect Dis* **2021,** 223, (3), 435-440.
13. Steinfeld, H.; Cho, M. T.; Retterer, K.; Person, R.; Schaefer, G. B.; Danylchuk, N.; Malik, S.; Wechsler, S. B.; Wheeler, P. G.; van Gassen, K. L.; Terhal, P. A.; Verhoeven, V. J.; van Slegtenhorst, M. A.; Monaghan, K. G.; Henderson, L. B.; Chung, W. K., Mutations in HIVEP2 are associated with developmental delay, intellectual disability, and dysmorphic features. *Neurogenetics* **2016,** 17, (3), 159-64.
14. Kerns, S. L.; Guevara-Aguirre, J.; Andrew, S.; Geng, J.; Guevara, C.; Guevara-Aguirre, M.; Guo, M.; Oddoux, C.; Shen, Y.; Zurita, A.; Rosenfeld, R. G.; Ostrer, H.; Hwa, V.; Dauber, A., A novel variant in CDKN1C is associated with intrauterine growth restriction, short stature, and early-adulthood-onset diabetes. *J Clin Endocrinol Metab* **2014,** 99, (10), E2117-22.
